# Primary Combined Hepatocellular-Cholangiocarcinoma: A Case of Underdiagnosed Primary Liver Cancer

**DOI:** 10.7759/cureus.18224

**Published:** 2021-09-23

**Authors:** Mohamad F Ayas, Saif Affas, Zayd Ayas, Momal Chand, Tarik Hadid

**Affiliations:** 1 Internal Medicine, Ascension St. John Hospital, Detroit, USA; 2 Basic Sciences, College of Natural Sciences, University of Texas at Austin, Austin, USA; 3 Pathology, Ascension St. John Hospital, Detroit, USA; 4 Oncology, Ascension St. John Hospital, Detroit, USA

**Keywords:** hepatocellular carcinoma (hcc), cholangiocarcinoma, combined hepatocellular-cholangiocarcinoma, trans-arterial radioembolization, liver tumor

## Abstract

Combined hepatocellular-cholangiocarcinoma (CHC) is a rare primary tumor of the liver. Histologically, it comprises components of both hepatocellular carcinoma (HCC) and cholangiocarcinoma (CC) but is associated with a worse prognosis. International guidelines regarding its management are scarce, with surgical management (major hepatectomy) being the treatment of choice. In this report, we present a challenging case of a 73-year-old male with primary CHC who was not a surgical candidate but underwent hepatic artery radioembolization instead.

## Introduction

Combined hepatocellular-cholangiocarcinoma (CHC) is a rare and aggressive primary hepatic malignancy consisting of combined phenotypical characteristics of both hepatocellular carcinoma (HCC) and cholangiocarcinoma (CC) [1]. The incidence of CHC is between 1-4.7% of all diagnosed primary liver tumors; however, it has been increasingly identified due to advances in immunohistochemistry (IHC) staining and genetic analysis [1,2,3], with a reported incidence of 0.4-14.2% across a number of studies. Despite being a distinct entity, there are no clear guidelines for managing these tumors, and surgical resection is the preferred treatment, with high curative rates [1,4,5]. However, in inoperable patients or those with tumor recurrence, nonsurgical management could be used, which includes transarterial chemoembolization (TACE), transarterial radioembolization (TARE), hepatic arterial infusion chemotherapy, and systemic chemotherapy [1]. We present a case of CHC in a 73-year-old male who was treated with TARE.

## Case presentation

**Figure 1:**
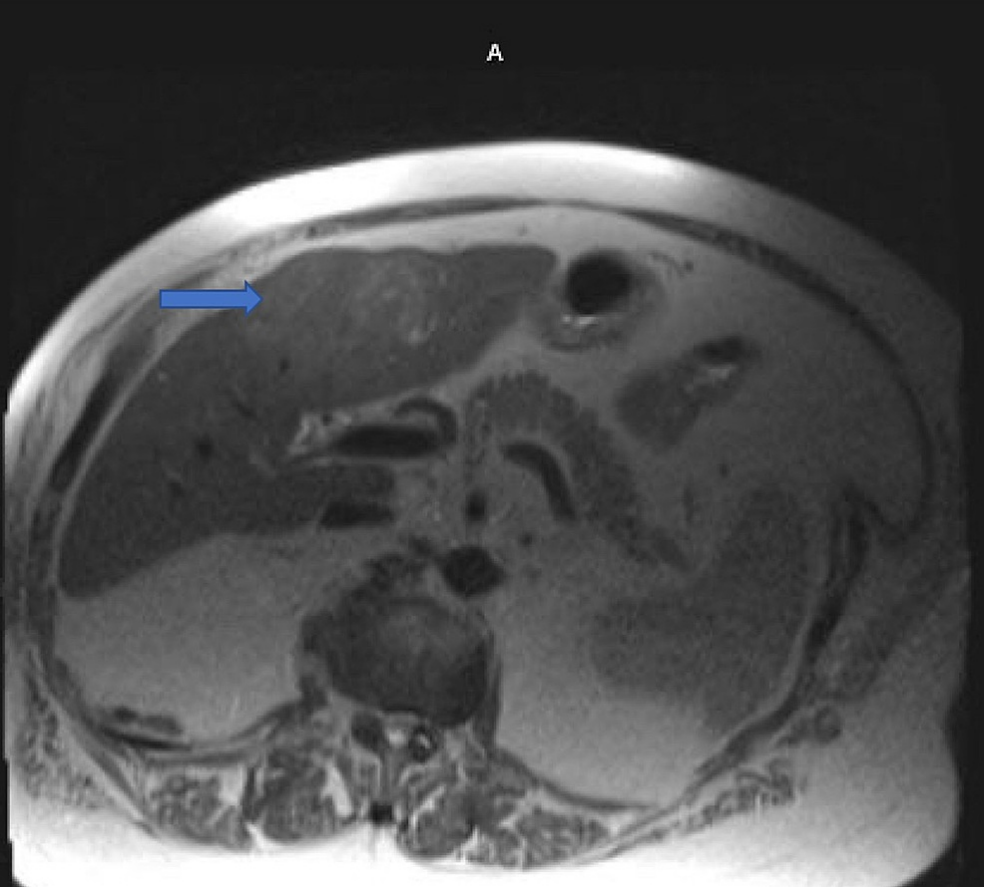
MRI showing a non-uniform enhancement of 6.4 x 8-cm mass in the left lobe of the liver (blue arrow) MRI: magnetic resonance imaging

A 73-year-old man with a past medical history of alcohol dependence presented with a two-week history of the right upper quadrant (RUQ) abdominal pain that was sharp in nature. The patient denied any nausea, vomiting, or recent change in bowel habits. He also denied any weight loss or any family history of malignancies. Vital signs were within normal limits. Abdominal examination showed tenderness in the RUQ, with a negative Murphy sign, and no hepatosplenomegaly was appreciated. Laboratory findings showed a hemoglobin of 9.9 gm/dl, an international normalized ratio (INR) of 1.31, total bilirubin of 2.2 mg/dl with a direct bilirubin of 0.7 mg/dl, alkaline phosphatase (ALP) of 334 IU/L, aspartate aminotransferase (AST) of 49 IU/L, and normal alanine aminotransferase (ALT) levels, with a negative hepatitis B and C panel. An abdominal ultrasound (US) was done, which showed an enlarged liver of 17.7 cm with two hypoechoic lesions within the right hepatic lobe. The patient then underwent a CT abdomen with intravenous (IV) contrast that did not demonstrate any liver lesions. Due to equivocal findings on the US and CT abdomen, the patient underwent an MRI of the abdomen with and without contrast, which showed a conglomerated 6.4 x 8-cm mass in the left lobe of the liver, possibly suggesting a hepatoma or neoplastic mass (Figure 1), with mild dilatation of the biliary ducts in the left lower lobe, and normal common hepatic and common bile ducts. Subsequently, an Oncology consult was requested.

**Figure 2:**
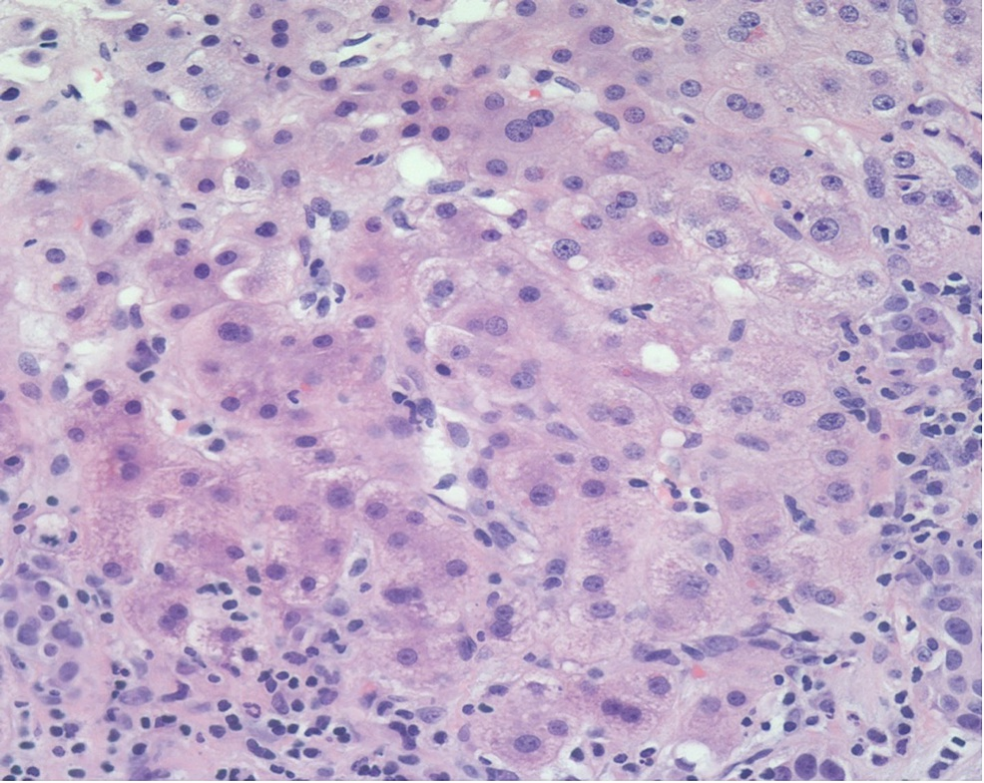
Hepatocellular carcinoma component, with loss of cellular architectural organization with eosinophilic cytoplasm

**Figure 3:**
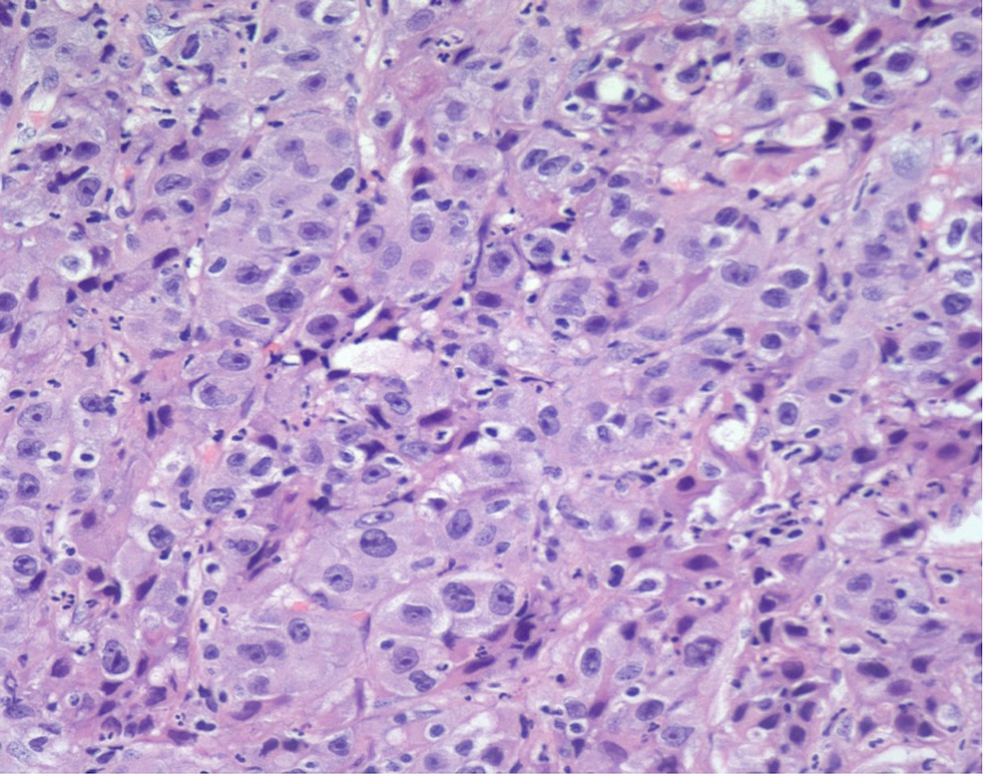
Cholangiocarcinoma component of the same tumor with vesicular cytoplasm; increased nuclear-to-cytoplasmic ratio

Tumor markers alpha-fetoprotein (AFP) and CA 19-9 were both negative. The patient then underwent a CT-guided liver biopsy. Pathologic analysis showed foci of tumor cells positive for hepatocyte antigen staining, with another focus consisting of closely packed tubules positive for CK7 and negative for hepatocyte antigen, consistent with CHC (Figures 2, 3). The patient at that time was deemed a poor surgical candidate and underwent nuclear arterial mapping followed by yttrium-90 radioembolization of the left hepatic artery. However, during the follow-up, MRI for restaging was done and showed increased right hepatic lobe tumor burden as well as complete venous thrombosis of the portal vein extending to the superior mesenteric vein. Due to the rapidly worsening performance status, the patient was not considered a candidate for further treatment and was later transferred to hospice.

## Discussion

CHC is a primary liver tumor with a vast histological spectrum and multiple classifications, making it a diagnostic challenge to pathologists, radiologists, and treating clinicians. It was first classified in 1949 by Allen and Lisa into three histological types; A, B, and C as described in Table 1 [1,2]. Subsequently, Goodman et al. re-classified CHC in 1985 into three separate entities: collision (type I), transitional (type II), and fibrolamellar (type III) (Table 1) [1,2,6]. Finally, in 2010, the World Health Organization (WHO) classified CHC into two types: classical and stem cell type, as described in Table 2 [3,7].

CHC should be suspected in patients with a discordant pattern between imaging and tumor markers [2,3]. To meet the diagnostic criteria, the sample must show indisputable evidence of hepatocellular and biliary differentiation. Thus, adequate tissue samples are needed to recognize the possible presence of both histopathologies. Furthermore, a core needle biopsy is preferred over fine-needle aspiration, in order to ensure adequate tissue sampling [2], and definitive diagnosis can only be made after a histological assessment of a representative biopsy and IHC staining [1].

According to Wang et al., a retrospective study of 642 patients with CHC between 2000-2014 was done and showed that the mean age at diagnosis was 62 years, with a male and Caucasian predominance [8]. Risk factors included alcoholism, male gender, hepatitis B virus (HBV), and hepatitis C virus (HCV) [2,9].

The consensus method for the treatment of CHC is hepatic resection with or without lymph node dissection [2], as it was observed that patients who did not undergo surgery had lower survival rates [10]. The one-year survival rate for CHC was found to be 41.9%, and the five-year survival rate was 17.7%, with a median survival rate of eight months [10]; however, there was a significant improvement in patients who underwent surgical resection [8]. There are conflicting study findings regarding the role of liver transplantation (LT) in CHC; however, most studies have documented positive outcomes, with a reported 40% five-year overall survival rate in some studies [11]. Moreover, one study has shown no difference in the overall three-year survival rate when comparing those with CHC undergoing surgical resection vs. those undergoing LT [12].

Although surgical resection is the only curative option, the use of TACE, TARE, and systemic chemotherapy can be considered for those with inoperable tumors [2]. The use of TARE has been minimally studied in the treatment of inoperable CHC. According to a study by Badar et al., a retrospective analysis involving 10 patients with CHC between 2013-2019 receiving TARE therapy, the median overall survival rate was 15.2 months [13]. Moreover, a similar study by Fowler et al. examined six patients with CHC who underwent TARE, and they had a median overall survival rate of 16 months [14]. Unfortunately, in our case, the patient had an overall survival rate of three months status post TARE therapy.

**Table 1:** Classification of CHC HCC: hepatocellular carcinoma; CC: cholangiocarcinoma; CHC: combined hepatocellular-cholangiocarcinoma

Allen and Lisa [1,2]	Tumor description	Goodman et al. [1,2,6]	Tumor description
Type A	HCC and CC at different sites but within the same liver	Type I	Collision tumor or an apparently coincidental occurrence of HCC and CC within the same liver
Type B	HCC and CC adjacent to each other	Type II	Transitional tumor in which there is a transition from HCC elements to CC elements
Type C	HCC and CC combined within the same tumor	Type III	Fibrolamellar tumor that resembles the fibrolamellar subtype of HCC but containing mucin-producing pseudoglands

**Table 2:** Classification of CHC by the World Health Organization WHO: World Health Organization; HCC: hepatocellular carcinoma; CC: cholangiocarcinoma; CLC: cholangiocellular; CHC: combined hepatocellular-cholangiocarcinoma

WHO classification [3,7]	Tumor description	
Classical type	Characterized by areas of typical HCC intermixed with CC with the presence of transition zones with intermediate cellular	
Stem cell type	Typical	Nest of mature hepatocytes surrounded by peripheral clusters of small cells exhibiting morphological and immunohistochemical characteristics of progenitor cells
	Intermediate	Cells with intermediate features between hepatocytes and cholangiocytes with immunohistochemical markers of both histological entities arranged in trabeculae, solid nests, or strands
	CLC	Cells morphologically mimicking cholangioles arranged in a tubular anastomosing (antler-like) pattern within a dense, sclerotic stroma and expressing progenitor/stem cell markers. Fibrolamellar tumor that resembles the fibrolamellar subtype of HCC but containing mucin-producing pseudoglands

## Conclusions

CHC is a rare primary liver tumor, and preoperative clinical diagnosis is extremely difficult, making it an often underdiagnosed entity. Histology and immunohistopathology are the only ways for a definitive diagnosis. Surgical hepatectomy with hilar lymph node resection is the only curative treatment; however, other treatment options such as TACE, TARE, and/or systemic chemotherapy should be considered in inoperable patients as described in our case.
